# Editorial: Equality, diversity and inclusive research for diverse rare disease communities

**DOI:** 10.3389/fpsyg.2023.1285774

**Published:** 2023-09-19

**Authors:** Andrew E. P. Mitchell, Federica Galli, Sondra Butterworth

**Affiliations:** ^1^Faculty of Health, Medicine and Society, University of Chester, Chester, United Kingdom; ^2^Department of Dynamic and Clinical Psychology, and Health Studies, Faculty of Medicine and Psychology, Sapienza University of Rome, Rome, Italy; ^3^ENRICH Research Facilitator, Swansea University, Swansea, United Kingdom

**Keywords:** rare diseases, rare mental health conditions, mental health, wellbeing, diversity in rare disease research, inclusive research, quality of life

## 1. Introduction

Rare diseases affect < 5 per 10,000 individuals, based on the European Union's definition (European Union, 2000). The Department of Health and Social Care (DHSC, 2021) published the UK Rare Diseases Framework, highlighting the government's dedication to enhancing the lives of the 3.5 million individuals affected by rare conditions in the UK (Rare Disease UK, 2000). A new framework has been established across the United Kingdom with four key priorities. These priorities include helping patients receive a final diagnosis quickly, increasing rare disease awareness among healthcare professionals, better coordination of care, and improving access to specialist care, treatments, and drugs (DHSC, 2021). The implementation must incorporate five supporting themes: patient voice, national and international collaboration, pioneering research, digital, data, technology, and broader policy alignment. The framework acknowledges the importance of comprehending the experiences of patients and their families and the value of collaboration between policymakers and service providers when designing services for those with rare diseases.

Research has shown that there needs to be more diversity in clinical trials, particularly for rare diseases (Gray et al., 2021). This can make it challenging to determine the safety and effectiveness of targeted treatments due to overlapping factors related to symptoms of these diseases and disparities in healthcare research (National Healthcare Quality and Disparities Report, 2021). Experts recommend increasing diversity in rare disease research to address these issues (Sharma and Palaniappan, 2021).

Living with a rare disease is physically and emotionally challenging, impacting individuals, families, relationships, finances, work-life, and future choices (Rare Disease UK, 2018). Uncertain prognoses, treatment pathways, lengthy diagnostic odysseys, and inter-acting symptoms impact self and identity (Tumiene and Graessner, 2021). There are over 7,000 rare diseases, many of which exhibit complex clinical characteristics (DHSC, 2021). These diseases often threaten an individual's life or result in chronic debilitation, accompanied by dysfunction across multiple bodily systems. It often involves emotional and mental health impacts such as low mood, anxiety, emotional exhaustion, and suicidal thoughts (Rare Disease UK, 2018).

All articles on the Research Topic mention the mental health and wellbeing of those with rare conditions and their families. It is also important to recognize that certain mental health disorders are classified as rare conditions and have their own cultural concepts of distress, as defined in the DSM-5 (American Psychiatric Association, 2013). For example, Khyâl Cap syndrome (Thornton, 2017), Kufungisisa (Backe et al., 2021), Clinical Lycanthropy (Guessoum et al., 2021), Capgras Syndrome (Shah et al., 2023), and Ekbom's syndrome (Ansari and Bragg, 2023) are rare conditions requiring equal attention and support for individuals and their families, both physically and emotionally.

## 2. Emotional and mental health impacts

The key priority of care coordination on the journey between pediatric and adult healthcare is explored by Alani. The absence of consistent care across these domains is a cause for concern among rare disease patients, irrespective of their diagnosis. The gap in frameworks and lack of coordination between services makes transitioning from pediatric to adult services challenging and can lead to a diminished sense of belonging, harming adolescents' mental health (Alani).

Also, the diagnostic journey for those with rare diseases presents a substantial obstacle for individuals and their families, primarily due to the inherent challenges associated with early and conclusive identification (Ng et al.), and it aligns with the key priority of early diagnosis. The extended duration of the diagnostic process may exert psychological pressure on patients, resulting in heightened levels of mental health conditions such as depression and anxiety. A key finding was that diagnosis can alleviate anxiety and stress.

The Undiagnosed Diseases Network International (UDNI) study found that scientific and medical institutions are making efforts to meet the needs of patients who have not yet been diagnosed (Taruscio et al.). The findings from the UDNI survey indicate that several challenges persist, including limited resources, disparities in service accessibility, international collaboration in data sharing, clinical research, and diagnostic proficiency and fit within the key priorities of making specialist care, treatments, and medications more accessible. One of the study's implications is implementing public health interventions incorporating relevant content into medical school curricula.

The key aspect of mental health and wellbeing was investigated by Dias et al. due to the burden of rare diseases on caregivers. In the context of Latin America, prevalent issues encompass physical pain (79%), poor sleep patterns (60%), and inadequate vitality to fulfill daily tasks (82%). Care provision also impacts mental health and wellbeing, as indicated by a significant proportion of individuals feeling lost (72%) and experiencing emotional isolation (68%).

The study conducted by Hughes et al. examined patients diagnosed with generalized myasthenia gravis (gMG) and focused on key priorities in diagnosis and treatment accessibility. The most prevalent concerns associated with gMG are financial considerations and mental wellbeing, with detrimental impacts on mental health observed across all stages of the condition. The findings suggest that monitoring mental health in underrepresented communities is needed to effectively address the adverse effects of depressive symptoms on the quality of life for individuals with gMG.

The final article by Imamatsu and Tadaka studied health behavior for preventing non-communicable diseases in the older adult population in Japan. The findings focused on the key priority of adopting mental health and wellbeing behavioral patterns associated with maintaining and promoting health, which was beneficial in preventing disease. The study indicates that adopting favorable health behaviors by older individuals reduces the risk of heart failure, diabetes, and cancer. However, older adults with limited financial resources face challenges engaging in beneficial health behaviors.

## 3. Conclusion

In summary, it is clear that rare disease communities have a range of experiences and barriers. Some of these challenges are common, while others are unique to individuals. It has emphasized that listening to the rare disease community is crucial to building trust. In addition to addressing psychosocial issues, the academic and clinical research community recognizes the need for changes from practitioners, researchers and the wider public. These changes should include factoring in the financial resources for research, outreach and engagement, treatment development and ensuring accessibility for all.

The studies published on this Research Topic cover various subjects relating to communities affected by rare diseases, such as their wellbeing, requirements, and improved treatments. The submissions covered a range of key priorities, including accelerating diagnosis, enhancing care coordination, and making specialist care, treatments, and medications more accessible, with mental health and wellbeing identified as key issue.

## Author contributions

AM: Writing—original draft. FG: Writing—review and editing. SB: Writing—review and editing.
